# Evaluating Laparoscopic Skills: Report on the Origami Crane Folding Competition Using Laparoscopic Instruments With Objective Criteria

**DOI:** 10.7759/cureus.72014

**Published:** 2024-10-21

**Authors:** Shunsuke Furukawa, Masatsugu Hiraki, Yosuke Hashimoto, Yusuke Noda, Hiroo Kanai, Masao Ichikawa

**Affiliations:** 1 Surgery, Hiramatsu Hospital, Ogi, JPN; 2 Laparoscopic Surgery, Community for Laparoscopic Training "Kaminote Challenge", Chiba, JPN; 3 Surgery, Saga University Faculty of Medicine, Saga, JPN; 4 Surgery, Shizuoka City Shizuoka Hospital, Shizuoka, JPN; 5 Urology, Anjo-kosei Hospital, Anjo, JPN; 6 Veterinary Medicine, Kanai Veterinary Surgery, Himeji, JPN; 7 Gynecologic Oncology, Nippon Medical School Chiba Hokuso Hospital, Chiba, JPN

**Keywords:** kaminote challenge, laparoscopic surgery, origami, paper crane, training

## Abstract

Purpose: To evaluate the importance of daily laparoscopic training using laparoscopic forceps to fold origami paper cranes (a traditional Japanese paper craft) and assess the performance of laparoscopic origami crane folding in an actual competition.

Method: A competition, named the "Kaminote Challenge World Championship," was used to evaluate the effectiveness of training. The participants folded the paper cranes using laparoscopic forceps. The judges evaluated the speed at which the paper cranes were folded and the quality of the completed cranes, using objective criteria.

Results: The competition was held twice, in 2022 and 2023, with 27 and 36 participants, respectively. The participants were surgeons, veterinarians, and students from Japan, Mexico, and Vietnam. The completion rate for folding a paper crane within seven minutes was 70.4% in 2022 (19/27) and 44.4% in 2023 (16/36). In the second competition, 75.0% (12/16) of the participants who completed the origami crane within seven minutes had practiced folding more than 1,000 cranes. Despite the competitive pressure, the top performers folded paper cranes with minimal deductions for quality and used the laparoscopic forceps precisely. The winners of the 1st prize in 2022 and 2023 completed the task in 2 min 46 s and 2 min 45 s, respectively, without any penalty.

Conclusions: Training by folding paper cranes using laparoscopic forceps is highly likely to lead to improved laparoscopic surgery skills. Such competitions may also be useful as an opportunity for individuals to demonstrate their forceps manipulation ability and maintain motivation.

## Introduction

Laparoscopic surgery, as well as robotic minimally invasive surgery, are considered major surgical procedures. Laparoscopic techniques have been used for various types of abdominal surgeries, including gastrointestinal and hepatobiliary pancreatic surgery, gynecological surgery, and urologic surgery. In addition, laparoscopic surgery has been applied in animal surgery [1]. However, mastery of surgical techniques is essential for safe and reliable surgery, and constant practice is desirable for this purpose. In comparison to robotic surgery, laparoscopic surgery might require more practice and time to master.

Recently, we reported the efficacy of laparoscopic training using origami (a traditional Japanese papercraft in which paper is usually folded by hand) by folding origami paper cranes using laparoscopic forceps [2,3]. This training is believed to improve hand-eye and left-right coordination, reduce tremors, and acquire delicate techniques [2,3]. There is also a report that the training has led to a significant reduction of actual operation time [4,5]. Various other approaches to laparoscopic training have been reported, such as suturing, ligating, excising, peg transfer, and rope race [6-9]. However, these training methods are monotonous, boring, and lack tangible training effectiveness. In contrast, origami crane training is considered very useful not only in terms of visual completion but also in terms of measurability, as parameters such as the time to completion and number of times achieved can be evaluated.

Since 2015, a community for laparoscopic training called "Kaminote Challenge" has been established in Japan with the aim of improving laparoscopic skills. The community's name “Kami-no-te” comes from the aspiration to master laparoscopic techniques, to such an extent that it reaches the realm of deities. “Kami” is the Japanese word for deities/supernatural beings. It is also a homonym for paper. “no” is just a postpositional particle in Japanese. “Te “ is the Japanese word for hand. We organized a laparoscopic skills competition (folding paper cranes using laparoscopic forceps) in which skills were compared in terms of speed and quality. In this report, we present the results and the participants’ achievements.

## Materials and methods

**Figure 1:**
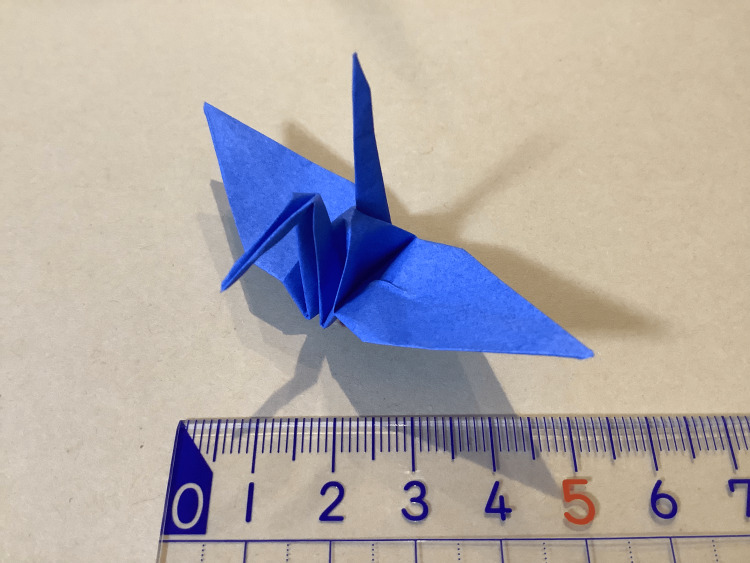
The completed folded paper crane

**Figure 2:**
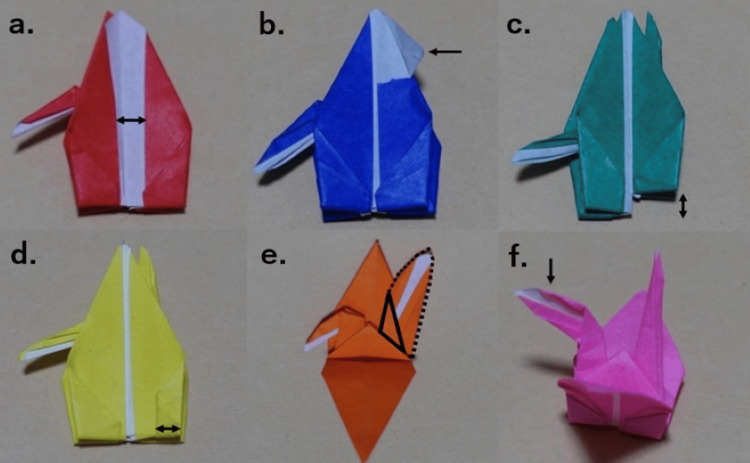
The time penalties in the competition (a) Gap between the wing >4 mm, +10 s. (b) Paper ripping >2 mm, +10 s. (c) Gap at the bottom >2 mm, +5 s. (d) Folded back >2 mm at the base of the wing, +5 s. (e) Extremely irregular tail, +5 s. (f)  Extremely irregular neck, +5 s

Participants were encouraged through social networking sites such as Facebook, X, and YouTube in 2022 and 2023. A competition named the "Kaminote Challenge World Championship" was held in December 2022 and December 2023. This competition was held to evaluate the effectiveness of training. Participants trained individually and competed in the competition. The organizers determined the approximate number of paper cranes each participant had folded during training and used this information to assign participants to preliminary groups. A laparoscopic box trainer was used to fold origami cranes. The video camera portrayed the inside of the box trainer on the screen. Laparoscopic forceps were used according to individual preferences, including LigaSure Maryland and Laparoscopic Maryland Dissecting Forceps, laparoscopic needle holders, etc. The origami paper used in this competition was white on one side, with various colors on the other. This origami paper is a standard paper and not a traditional paper made of washi. The color of the origami paper was chosen according to each participant's preference. The dimensions were 75.0 × 75.0 × 0.05-0.12 mm. The time required to complete the paper crane test was measured. The competition was a hybrid of on-site and online competition. The time limit was set at seven minutes. In addition to the speed at which the paper crane was folded, the quality of the paper crane was evaluated by judges. The completed folded paper crane is shown in Figure 1. Time penalties were imposed for completed paper cranes with misaligned folds or damaged paper. The time penalties are shown in Figure 2. The following penalties were applied: gap between the wing >4 mm, +10 s; paper ripping >2 mm, +10 s; gap at the bottom >2 mm, +5 s; folded back >2 mm at the base of the wing, +5 s; extremely irregular tail, +5 s; and extremely irregular neck, +5 s. The top performers from the preliminary rounds advanced to the final, where they competed again. A video of the folding paper crane is provided in Video 1.

**Video 1:** A video of the training by author YH

## Results

**Figure 3:**
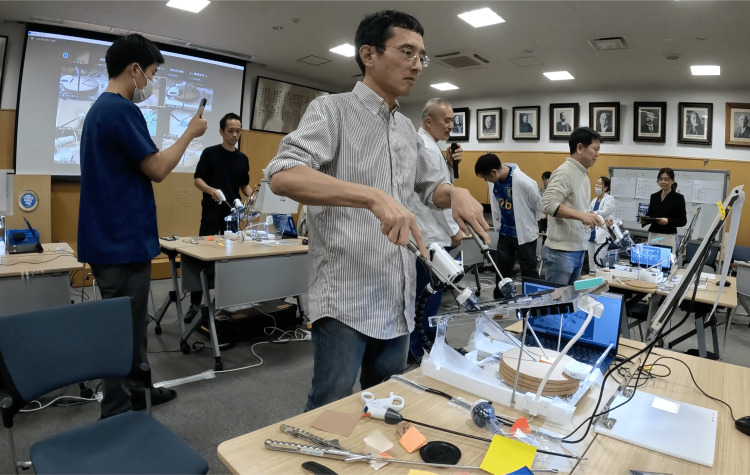
Scene of the competition in 2023

This competition was held in December 2022 and December 2023, with 27 and 36 participants, respectively. The breakdown of participants was as follows: 25 doctors, one veterinarian, and one medical student in 2022; 23 doctors, three veterinarians, and 10 medical students in 2023. Almost all the participants were from Japan. One person from Mexico and another from Vietnam participated in 2022, and one person from Mexico participated in 2023. The rate of completion within seven minutes was 70.4% in 2022 (19/27) and 44.4% in 2023 (16/36). In the second competition, among participants who had practiced folding more than 1,000 cranes before the competition, 75% of participants completed the origami crane within seven minutes (12/16). The actual competition scene for 2023 is shown in Figure 3. The results for the top rankers in each competition are shown in Tables 1-2. The winning times in 2022 and 2023 were 2 min 46 s (without penalty) and 2 min 45 s (without penalty), respectively.

**Table 1:** The result of the 1st competition of laparoscopic folding paper crane in 2022 *Current institution, **Previous institution ※Endoscopic Surgical Skill Qualification System from Japan Society for Endoscopic Surgery

Rank	Participant	Affiliation	Specialty	Number practiced	Actual time	Penalty time	Total time	Place of participation	Qualification※
1st	1	Shizuoka City Shizuoka Hospital	Gastrointestinal surgery	11,830	2’ 46’’	00’’	2’ 46’’	on-site	Yes
2nd	2	Yamaguchi University	Gastrointestinal surgery	2,500	3’ 09’’	00’’	3’ 09’’	online	No
3rd	3	Kanai Veterinary Surgery	Veterinarian	6,602	3’ 30’’	00’’	3’ 30’’	on-site	No
4th	4	PL General Hospital	Urology	12,000	3’ 35’’	00’’	3’ 35’’	on-site	No
5th	5	Central Japan International Medical Center	Gastrointestinal surgery	1,000	3’ 36’’	00’’	3’ 36’’	online	No
6th	6	Saga University*	Gastrointestinal surgery	3,000	3’ 59’’	10’’	4’ 09’’	online	Yes
7th	7	Asahikawa Medical University	Resident	896	3’ 59’’	15’’	4’ 14’’	online	No
8th	8	Koyama Memorial Hospital	Gynecology	1,200	3’ 56’’	20’’	4’ 16’’	online	No
9th	9	Anjo-kosei Hospital	Urology	3,000	4’ 21’’	00’’	4’ 21’’	online	No
10th	10	Japanese Red Cross Society Karatsu Red Cross Hospital**	Gastrointestinal surgery	600	4’ 28’’	05’’	4’ 33’’	online	No

**Table 2:** The result of the 2nd competition of laparoscopic folding paper crane in 2023 *Current institution, **Previous institution ※Endoscopic Surgical Skill Qualification System from Japan Society for Endoscopic Surgery

Rank	Participant	Affiliation	Specialty	Number practiced	Actual time	Penalty time	Total time	Place of participation	Qualification※
1st	1	Yamaguchi University	Gastrointestinal surgery	3,500	2’ 45’’	00’’	2’ 45’’	online	No
2nd	2	Shiroishi Kyouritsu Hospital**	Gastrointestinal surgery	2,200	2’ 58’’	05’’	3’ 03’’	online	No
3rd	3	PL General Hospital	Urology	24,000	3’ 10’’	00’’	3’ 10’’	on-site	No
4th	4	Shizuoka City Shizuoka Hospital	Gastrointestinal surgery	13,870	3’ 27’’	00’’	3’ 27’’	on-site	Yes
4th	5	Kanai Veterinary Surgery	Veterinarian	7,313	3’ 27’’	00’’	3’ 27’’	online	No
6th	6	Nagano Red Cross Hospital	Urology	400	4’ 25’’	10’’	4’ 35’’	on-site	No
7th	7	Ukyo Veterinary Clinic	Veterinarian	700	4’ 42’’	00’’	4’ 42’’	online	No
8th	8	Saga University*	Gastrointestinal surgery	3,050	5’ 06’’	15’’	5’ 21’’	on-site	Yes

## Discussion

This report demonstrates a community competition in which participants folded paper cranes using laparoscopic forceps, competing for both speed and quality. Surprisingly, top-level participants completed the task in a very short time (<3 min) with a high degree of quality. Most of the top-level participants practiced folding paper cranes more than 1,000 times (Tables 1-2). In addition, the participants who practiced the most had practiced 24,000 times. While some participants achieved top ranks with only about 500 practice sessions, it was shown that daily practice and folding many cranes were necessary to meet the seven-minute cutoff time. It is possible that the online participants had an advantage because they were able to participate in their usual environment rather than on-site. The top participants tended to have fewer unnecessary movements (Video 1). While training time and repetition are important, the quality of training might be also important, such as the reduction of unnecessary movements and the formulation of procedures. Completing a single origami crane is a considerable feat, and even surgeons who perform laparoscopic surgery take 80-120 minutes for the first attempt [3], often without completing the task. Thus, these results can be attributed to the fact that they had been practicing and improving their skills.

**Figure 4:**
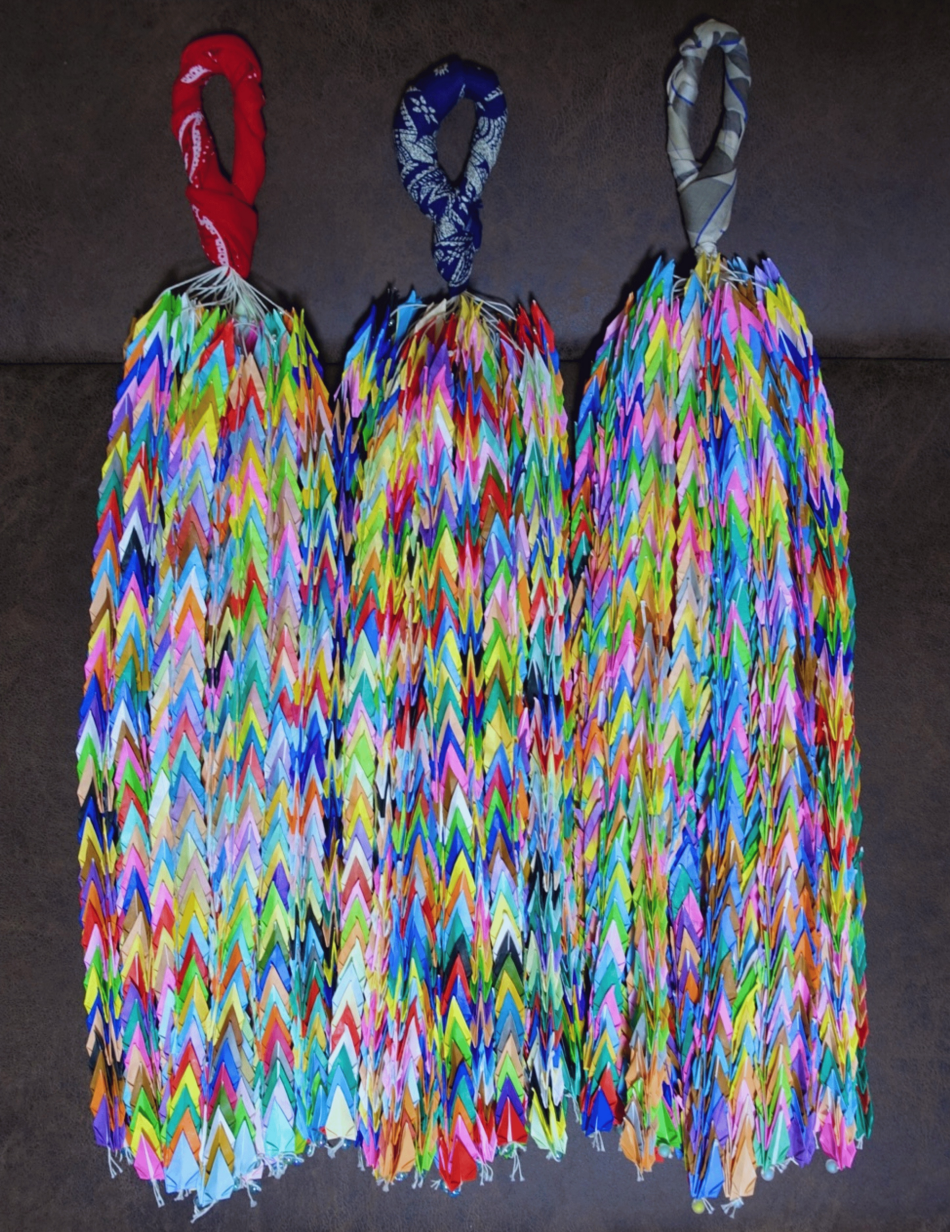
Three sets of 1000 paper cranes made by author SF

This community, named "Kaminote Challenge," was established with two aims. The first aim is to fold paper cranes laparoscopically, competing and stimulating growth between community members, thus resulting in high laparoscopic skills. The second aim is to improve members’ skills by creating 1,000 cranes for training. We set the goal of folding 1,000 paper cranes because there is a tradition of sending 1,000 paper cranes to pray for early recovery from illness or injury, to pray for the recovery of disaster-stricken areas, or to pray for victory in a sports game in Japan. We believe that folding a large number of paper cranes, such as 1,000 paper cranes, as part of laparoscopic training, not only prays for the early recovery of the patient and the success of the surgery but also enhances the surgeon’s own skills (Figure 4). This competition has been held for two years with the objective of assessing the proficiency in laparoscopic community. The competition has led to the improvement of skills and the formation of a broad community.

Currently, the opportunities for laparotomy have drastically reduced, and minimally invasive surgeries such as laparoscopic and robotic surgeries have become standard surgical procedures. In general, however, minimally invasive surgery tends to prolong the operative time relative to laparotomy [10-14]. Even if the wound in laparoscopic surgery is smaller than that in laparotomy, the patient's burden from general anesthesia and surgery may not be reduced if the operative time is increased. Therefore, surgeons still need to improve their skills to achieve better patient outcomes. In fact, surgeons trained in laparoscopic origami crane folding can do this in as little (or less) time as it takes to fold a paper crane by hand. Typically, folding a crane by hand takes at least about three minutes. The results of this training could lead to a reduction in the operation time in laparoscopic surgery without degrading the quality of the actual surgery. The process of folding paper cranes includes clutching, pulling, holding using forceps, and placing forceps in gaps. Although techniques and training in sutures and ligation are also important, the majority of surgical techniques require coordinated and delicate left-right movements, and this training will help develop flexible forceps manipulation and movements. It would be true that laparoscopic training is useful for surgeons of all backgrounds, whether they work in hospitals with high or low surgical volumes, and whether they are young or senior doctors. It is generally thought that mastering surgical techniques requires a certain number of surgeries [15]. However, it is possible to improve surgical techniques without having to perform many surgeries by practicing sufficiently before actually performing the surgery. The paper crane training method has the potential to contribute to this. If the learning curve for surgeons can be reduced, this will be of great benefit to patients.

Another advantage of this training is that young surgeons, even medical students, can easily practice in an environment where they are allowed. It is important that it is low cost and therefore easily accessible in ongoing training [16,17]. The cost of this training is very low compared to that of standard laparoscopic training [4,18]. Laparoscopic suturing training requires many needles and threads; conversely, laparoscopic training using origami is sufficiently accomplished with just paper. However, this training has different aspects than training in needle and thread suturing. Furthermore, it should be kept in mind that separate training must be done to master the technique of needle and thread suturing. Additionally, while 3D printing and virtual reality simulators are becoming more common, their high cost and difficult accessibility may make them unsuitable for routine training. The medical students and residents who have participated in this training so far have been motivated by the anticipation of competing. Young surgeons may not have sufficient opportunities to perform these surgeries. Thus, training and competition may be important for providing incentives and maintaining motivation. Additionally, to motivate participants in the second competition, non-fungible tokens (NFTs) were issued as proof of participation for those who requested them, recording the time taken to fold an origami crane. These NFTs, based on blockchain technology, serve as immutable records that can be verified by anyone. To view this, go to the website: https://pool.pm/search/ and enter the number 992b90b0aabc54fafb6464f40694ea1c6a46436ce647d945570b1043. This system was well-received by the participants. Our community hopes that numerous surgeons will be interested and that many surgeons will participate in this competition.

This study has several limitations. First, the participants in this training may be subject to selection bias. Those interested in this training might already be generally motivated toward various types of training, so improvements in laparoscopic skills may not be solely attributable to this training. However, previous study has shown that laparoscopic training using origami possibly led to improvements in laparoscopic surgical skills and surgical outcomes compared to common laparoscopic surgery training of techniques such as suturing and ligation [4,5]. Second, the transition to robotic surgery could reduce opportunities for laparoscopic surgery. However, it has been reported that a trainee’s baseline laparoscopic skills correlate with certain baseline robotic skills [19]. Since robotic surgery training is not easily accessible, the importance of laparoscopic training remains high even in this context. Additionally, in developing countries, considering the lack of widespread adoption of robotic technology and its high cost, laparoscopic surgery still has a definite role [20]. Third, a shorter time to fold paper cranes does not necessarily correlate with better surgical skills. It is crucial not only to view the task of folding cranes as a time-based exercise but also to carefully consider how the forceps action involved can be applied to different aspects of surgery. Fourth, there is a lack of outcome-based data on how this training specifically impacts surgeons at different stages of their careers. Previous reports have indicated that laparoscopic training using origami contributes to improvements in laparoscopic skills and surgical outcomes for young surgeons [4,5]; however, the evidence of this training is still under evaluation, and the impact on accomplished surgeons remains unclear.

## Conclusions

This study evaluated the importance of daily laparoscopic training using laparoscopic forceps in folding origami paper cranes and assessed the performance of laparoscopic origami crane folding in actual competitions. Daily training in folding paper cranes using laparoscopic forceps is low cost and easily accessible, and it is likely to improve laparoscopic surgery skills, even under the high-pressure conditions of a competition. Moreover, this form of competition can serve as a valuable chance to showcase one's proficiency in handling forceps to doctors and patients from various backgrounds. It can also help maintain motivation by connecting with peers who share similar objectives.
